# Exploring the Influence of P3HT on PTCA Crystallization and Phase Behavior in Thin Films

**DOI:** 10.3390/nano13222918

**Published:** 2023-11-08

**Authors:** Pallavi Kumari, Barbara Hajduk, Henryk Bednarski, Paweł Jarka, Henryk Janeczek, Mieczysław Łapkowski

**Affiliations:** 1 Centre of Polymer and Carbon Materials, Polish Academy of Sciences, 34 Marie Curie-Skłodowska Str., 41-819 Zabrze, Poland; bhajduk@cmpw-pan.pl (B.H.); hbednarski@cmpw-pan.pl (H.B.); hjaneczek@cmpw-pan.pl (H.J.); mieczyslaw.lapkowski@polsl.pl (M.Ł.); 2 Department of Engineering Materials and Biomaterials, Silesian University of Technology, 18a Konarskiego Str., 41-100 Gliwice, Poland; pawel.jarka@polsl.pl; 3 Department of Physical Chemistry and Technology of Polymers, Faculty of Chemistry, Silesian University of Technology, M. Strzody 9, 44-100 Gliwice, Poland

**Keywords:** optical properties, organic semiconductors, spectroscopic ellipsometry, thermal properties, thin films

## Abstract

The thermal properties and alignment of crystallinity of materials in thin films play crucial roles in the performance and reliability of various devices, especially in the fields of electronics, materials science, and engineering. The slight variations in the molecular packing of the active layer can make considerable differences in the optical and thermal properties. Herein, we aim to investigate the tuning of the physical properties of a blended thin film of n-type small organic molecules of perylene-3,4,9,10-tetracarboxylic acid (PTCA-SMs) with the mixing of the p-type polymer poly(3-hexylthiophene) (P3HT). The resulting thin films exhibit an enhanced surface crystallinity compared to the pristine material, leading to the formation of long crystallites, and these crystallites are thermally stable in the solid state, as confirmed by X-ray diffraction (XRD), atomic force microscopy (AFM), and thermal analysis using variable-temperature spectroscopic ellipsometry (VTSE) and differential scanning calorimetry (DSC). We believe that the crystalline structure of the obtained P3HT/PTCA-SMs blends is a combination of edge-on and face-on orientations, which enable the potential use of this material as an active layer in organic electronics.

## 1. Introduction

Organic semiconducting thin films (OSTFs) have garnered significant global attention due to their potential applications as an active layer in cutting-edge electronic and optoelectronic devices [1,2,3]. These materials have also represented unique physical qualities, which include mechanical flexibility, compatibility with plastic substrates, and the ability to modify their optoelectronic properties synthetically [4,5]. The major impediment in the development of organic electronics is the necessity to precisely control the crystalline packing and nano/microstructure of electron-donor and -acceptor materials within thin films. The optimization of these nano/microstructures with appropriate properties is made possible by carefully managing how donor/acceptor molecules assemble together on a surface, forming crystallites and ultimately determining the morphology of thin films. Extensive research has been conducted to improve the optoelectronic performance of organic semiconductors by manipulating their crystallinity and shape [6]. For example, Chen et al. demonstrated in 2013 that combining two conjugated polymers as additives at a ratio of 1:1 with the small-molecule 6,13-bis(triisopropylsilylethynyl) pentacene resulted in the precise manipulation of thin-film structure and polymorphism, eventually leading to unique crystal structures that enhanced the long-range order and charge transport [7]. Similarly, Bi et al. also investigated the use of the polymer P3HT as an agent in combination with DPP-based materials, which resulted in the formation of well-aligned, highly crystalline crystals and a significant improvement in the performance of organic field-effect transistors [8]. Thus, thin films composed of a combination of n-type small organic molecules and p-type conjugated polymers have garnered substantial attention in the research due to their potential for applications in cost-effective, expansive, and flexible optoelectronic devices [9,10]. P-type conjugated polymers have a pivotal role in the development of various optoelectronic devices ranging from field effect transistors [11], solar cells [12], sensors [13,14], and organic light-emitting diodes [15]. Among these conjugated polymers, regioregular poly(3-hexylthiophene) P3HT stands out as one of the extensively used and studied p-type conjugated polymers due to its ease of synthesis, solution processing, and excellent electrical and optical characteristics [16,17]. Moreover, the electrical and morphological properties of P3HT can be tailored by introducing small molecules [18]. Thus, in this study, we employ the simple organic molecule perylene tetracarboxylic acid (PTCA-SMs) in combination with P3HT.

Perylene-3,4,9,10-tetracarboxylic dianhydride (PTCDA) and its derivatives represent a highly promising class of n-type materials exhibiting excellent photophysical and thermal stability properties [19,20]. These materials are particularly well-suited for their application in the development of electronic and optoelectronic devices due to their accurate contact between the molecules through π-stacking, electrostatic forces, and H-bonding which allows efficient charge propagation. However, why optoelectronic devices constructed using mixtures of polythiophenes and perylene derivatives often fall short of their expected performance still needs to be understood. The performance of OSTFs is intricately linked to their internal morphology, encompassing factors such as the phase separation between donor and acceptor domains, crystallinity, crystallite orientation, and vertical distribution of the respective domains [21]. Furthermore, the molecular and internal structures of OSTFs are profoundly influenced by their physical parameters [22]. In pursuit of enhancing charge propagation and electron transport within these organic semiconductors, we focus on developing more organized nanomaterials at first. These materials should exhibit improved conductivity, enabling an intentional charge movement across the electrodes.

This article delves into an exploration of the intricate physical interaction between blended PTCA-SMs and P3HT, highlighting their influence on the crystallization of semiconducting molecules and the phase behavior of the donor and acceptor mixture. As materials-based technologies advance, the need for novel and more precise approaches to characterize materials is becoming increasingly apparent. Thus, in this study, we use ellipsometry as a highly sensitive and non-invasive tool for investigating thin films composed of small molecules, conjugated polymers, and their blends, specifically to determine their optical and thermal properties. We successfully determine the critical parameters, such as the glass transition temperature (T_g_), cold crystallization temperature (T_CC_), and crystallization melting temperature (T_CM_), by constructing a comparative diagram utilizing ellipsometry and differential scanning calorimetry (DSC) data [23,24,25]. The temperature values associated with these thermal transitions, as revealed by the raw ellipsometric data [26,27,28], are corroborated with DSC measurements, serving as a reference technique. Furthermore, our analysis extends to the identification of thermal transitions through temperature-dependent variations in multiple physical parameters, including changes in the film thickness [d(T)] and refractive index [n(T)]. These thermal transition temperature values are discerned by locating the intersections of linear fits to the data points on graphs [29,30,31,32]. To achieve a comprehensive understanding of the film’s microstructure, we complement our ellipsometry and DSC investigations with X-ray diffraction (XRD) and atomic force microscopy (AFM) studies, delving into both the bulk and surface characteristics of the P3HT-PTCA-SMs blend films.

## 2. Materials and Methods

### 2.1. Materials

All chemicals, including 2-bromo 5-Iodo 3-hexylthiophene, 1,3-bis(diphenylphosphino) propanenickel(II) chloride [Ni(dppp)Cl_2_], and isopropymagnesium chloride, were purchased from Sigma-Aldrich Pvt. Ltd., Poznań, Poland, and were used as received. Perylene-3,4,9,10-tetracarboxylic dianhydride, potassium hydroxide (KOH), and solvents chloroform (CHCl_3_), tetrahydrofuran (THF), methanol, and dimethylformamide (DMF) were obtained from Merck (Warszawa, Poland) and were used as received. All other chemicals were used without further purification unless otherwise mentioned. For the purification of PTCA, deionized water was obtained from a Polwater system (Labopol Polwater; Kraków, Poland).

### 2.2. Synthesis of P3HT

The typical synthesis procedure of P3HT was as follows [33]. A total of 1 g (5 mmol) of 2-bromo-5-iodo-3-hexylthiophene monomer was placed in a dried Schlenk flask continuously purged with a highly pure argon atmosphere. A total of 25 mL of dry THF was added into the Shlenk flask, then treated with 1 equiv. of isopropylmagnesium chloride at 0 °C, and the solution was stirred for 30 min at 0 °C. Then, the solution was cooled to room temperature, followed by the addition of 0.4 mol % of Ni(dppp)Cl_2_ in 10 mL of THF and stirred at room temperature for 24 h. The reaction mixture was quenched by 1 mL of aqueous HCl (5 M), then precipitated in methanol to receive a purple solid product, which was filtered and washed with excess methanol. The oligomer or low-molecular-weight fraction was removed by the successive washing of the product with hexane and dried under vacuum to yield (50% yield). GPC: Mn = 9000 g/mol, PDI = 1.47. The structural characterization of P3HT was achieved by H^1^NMR spectroscopy. The regioregularity of P3HT was 94%, calculated by NMR (CDCl_3_)—6.98 (1H, s, CHaro), 2.8 (2H, t, CH_2_–CH_2_)_4_–CH_3_), 1.70 (2H, m, CH_2_–CH_2_–(CH_2_)_3_−CH_3_)), 1.44–1.35 (6H, m, CH_2_–CH_2_–(CH_2_)_3_–CH_3_)), and 0.91 (3H, t, CH_2_–(CH_2_)_4_–CH_3_.

### 2.3. Synthesis of PTCA-SMs

The PTCA was prepared by the hydrolysis of PTCDA, as previously reported [34,35]. In 100 mL of deionized water, 500 mg of PTCDA was dissolved. A total of 0.35 g of KOH was added after 20 min of ultra-sonification. Then, the mixture was rapidly placed into an 80 °C water bath. The mixture was agitated for one hour to create a translucent yellow-green solution. The solution was allowed to cool and then filtered so as to remove trace amounts of unreacted PTCDA, if any, and then 1 mL of HCl was added dropwise to the solution at room temperature. High-water immiscibility PTCA was precipitated from the solution. The addition of 1 mL of HCl continued until the green fluorescence of the solution completely vanished. For an additional hour, this PTCA dispersion was swirled at room temperature. Then, the dispersion was filtered using filter paper with a pore size of 0.2 µm, and it was repeatedly washed in water until the PH of the solution was neutral. The solid PTCA powder in a reddish orange color was vacuum-dried at 60 °C. The PTCA structure was characterized using FTIR (Figure S2). The peaks at 1778 and 3443 cm^−1^ corresponded to the C=O and O-H vibrations of PTCA, respectively, with a yield = 99%. FTIR (KBr) Vmax 3519, 1778, 1687, 1584, 1437, 1276 and 845 cm^−1^. 

### 2.4. Thin-Film Preparation 

Thin films of P3HT, PTCA-SMs, and P3HT:PTCA-SMs were prepared using the drop-casting method on a microscopic glass substrate. For the individual P3HT and PTCA-SMs films, each material was dissolved in anhydrous CHCl_3_ and DMF, respectively, at a concentration of 5 mg/mL. The deposition of P3HT was conducted at room temperature, while the PTCA-SMs were deposited at 70 °C. The P3HT:PTCA-SMs blend was prepared by mixing P3HT and PTCA separately in CHCl_3_ and DMF vials, respectively, each at a concentration of 5 mg/mL, and then stirring the mixture for 24 h. The resulting stirred solution was deposited at 60 °C and the thickness of the film was measured using variable-angle spectroscopic ellipsometry. The thicknesses of PTCA-SMs, P3HT, and P3HT/PTCA-SMs-blend films were 150, 83, and 71 nm, respectively.

### 2.5. Characterization

#### 2.5.1. Nuclear Magnetic Resonance (NMR) Spectroscopy 

The ^1^H NMR spectra were recorded at 25 °C on a NMR Spectrometer Bruker Avance II 600 MHz Ultrashield Plus instrument (Karlsruhe, Germany). The ^1^H NMR chemical shifts (δ) were reported in parts per million (ppm).

#### 2.5.2. Fourier-Transform Infrared (FT-IR) Spectroscopy 

The PerkinElmer Spectrum Two (Waltham, MA, USA) spectrometer was used to conduct infrared (IR) spectroscopy. It is outfitted with a universal attenuated total-reflectance (UATR) (single-reflection diamond) module.

#### 2.5.3. Size-Exclusion Chromatography 

The molecular weight and polydispersity of the polymer were analyzed by a size-exclusion chromatography analysis, which was calibrated using linear polystyrene standards (580 to 1,390,000 g/mol), at a flow rate of 0.8 mL/min at 30 °C. An MDS RI Detector for the differential refractometer was included with the size-exclusion chromatography using a HPLC 1260 Infinity system (Agilent Technologies, Santa Clara, CA, USA).

#### 2.5.4. Spectroscopic and Variable-Temperature Ellipsometries 

Ellipsometric measurements were performed with a SENTECH SE 850E ellipsometer, operated using spectroscopic ellipsometry software version SpectraRay3. This ellipsometer is capable of operating within the wavelength range of 240–2500 nm. The absorption and optical coefficients (refractive index (n) and extinction coefficient (k)) of the films were determined using transmission and variable-angle ellipsometry modes. To perform the transmission measurement, a dedicated transmission holder was used with the goniometer angle fixed to 90°. The ellipsometric angles Ψ (the amplitude) and Δ (phase difference) were recorded at a 10° interval within the angular range of 40–70°. Variable-temperature measurements were conducted within a vacuum chamber with a pressure of (approximately) 10^−1^ Torr. Temperature variations were controlled using an INSTEC mK 1000 controller, and the electrical heater was cooled through a liquid nitrogen pump. Similar to our previous studies [36,37,38], we followed a standard methodology where the maximum set temperature depended on the specific characteristics of the material under investigation. Each sample was annealed until it reached a temperature close to its melting point followed by gradually being cooled down to −50 °C, with a cooling rate of 100 °C/min. Spectra were acquired within the spectral range of 240–930 nm at 10 s intervals with a heating rate of 2 °C/min.

#### 2.5.5. Differential Scanning Calorimetry (DSC) Analysis 

DSC measurements were performed on P3HT, PTCA-SMs, and a blend of P3HT and PTCA-SMs materials. The measurements were conducted using DSC Q2000 (TA Instruments, Newcastle, DE, USA), with aluminum sample pans. The thermal characteristics of the samples were obtained under a nitrogen atmosphere with a gas flow of 50 mL/min. The instrument underwent a calibration using high-purity indium standards, with cooling and heating rates of 20 °C/min.

#### 2.5.6. Atomic Force Microscopy (AFM) Analysis

The surface morphologies of the thin films from P3HT, PTCA-SMs, and a blend of P3HT and PTCA-SMs materials were studied by atomic force microscope (AFM) using Park Systems XE 100 with dedicated XEI Softwares 5.2 Build 1 (Suwon, Republic of Korea). This software enables the processing of images and analyzing surface roughness parameters. The microscopic measurements were conducted in the non-contact mode using silicon AFM probes with a tip radius <10 nm.

#### 2.5.7. X-ray Diffraction (XRD) Analysis 

The X-ray diffraction patterns were obtained using the D8 Advance diffractometer (Bruker, Karlsruhe, Germany) with a Cu-Kα cathode (λ = 1.54 Å). Due to the high film thickness of the samples, around 1000 nm, the classic Bragg–Brentano geometry measurement was applied. The scan rate was 1.2°/min with a scanning step of 0.02° in the range of 2° to 60° (2Θ) (dwell time: 1 s). Background subtraction, occurring from air scattering, was performed using the DIFFRAC.EVA program.

## 3. Results and Discussion

**Scheme 1:**
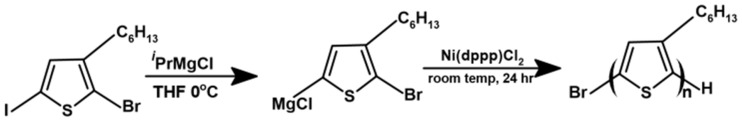
Synthesis of P3HT.

**Scheme 2:**
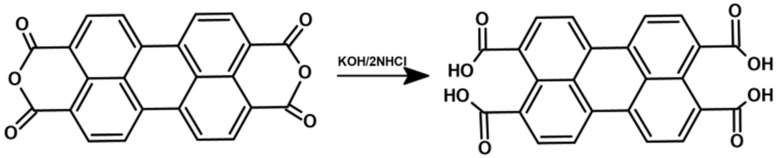
Synthesis of PTCA-SMs.

**Figure 1:**
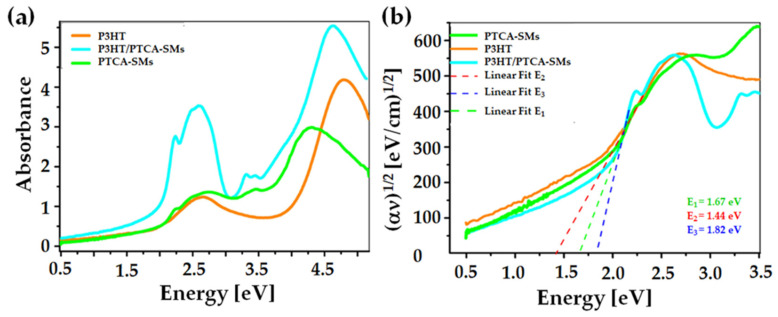
(**a**) Absorption spectra of P3HT, PTCA-SMs, and their blend films; (**b**) Tauc graphical method of energy gap determination.

Scheme 1 and Scheme 2 illustrate the straightforward route for the synthesis of P3HT and PTCA [33,34,35]. The structural characterization of P3HT was achieved by ^1^H NMR (Figure S1). Additionally, the molecular weight of P3HT was determined by the GPC analysis. For PTCA-SMs, a structural characterization was accomplished through FT-IR spectroscopy (Figure S2). The absorbance spectra of P3HT, PTCA-SMs, and their blends were deposited onto a microscope glass (thickness: 0.1 mm) (Figure 1a). The spectra were obtained using the ellipsometer transmission mode, in the 240–2500 nm wavelength range. The absorbance was calculated using the following relation [39]:

(1) $$Abs=\ln(\frac{1}{T})$$

where *T* is the transmission of the sample.

The P3HT spectrum consisted of two broad bands with maxima at around 2.63 and 4.83 eV. The first band was notably broad, spanning from 1.85 to 3.65 eV, while the second band began at around 3.86 eV and extended beyond the recorded spectral range. Both of these visible bands contributed to π–π* electronic transitions [40]. Furthermore, the PTCA-SMs spectrum had three main bands with maxima recorded at around 2.71, 3.45, and 4.32 eV. The first two absorption bands that were assigned to π–π* transitions and the third band originated due to the charge transfer from the spz mixing orbital to the electron system of the macrocyclic ring of the PTCA-SMs [41]. In the first and second bands, the visible vibronic structure originated from electron–phonon interactions resulting from the vibrations of stretching aromatic rings. The spectrum of the P3HT:PTCA-SMs-blend film also contained three main bands with maxima recorded at around 2.60, 3.40, and 5.76 eV. The vibronic structures of the spectra were also visible on the first two of them.

To measure the optical energy bandgap E_g_ of the thin films, the Tauc method was used and based on the extrapolation of the linear fit at the location of the fastest function increment (Figure 1b). The Tauc relation is described by the following equation (the implication is true for E > E_g_) [42,43]:

*α* ∝ (*E* − *E_g_*)^2^ (2)

This method, usually used for inorganic semiconductors, was also applied for amorphous polymers [44,45]. The obtained values for the energy gap were included in the range of 1.4–1.9 eV. Their values were approximately (1.67 ± 0.02) eV for the PTCA-SMs film, (1.44 ± 0.02) eV for P3HT, and (1.82 ± 0.02) eV for the P3HT:PTCA-SMs-blend layer. The film thickness had no impact on the band gap of the thin films of the material (Figure S3). For the optical coefficient measurement, the materials casted onto opaque glass were measured using a standard ellipsometry variable angle mode. The ellipsometric angles Ψ and Δ were obtained within a 40–70° angle range, every 5°. The optical coefficients of P3HT were obtained using an ellipsometric model of the biaxial anisotropic layer (Figure S4a), where the model in the “xy” plane included three Leng–Lorentz oscillators and in the “z” direction; one Leng–Lorentz oscillator was used. Five Tauc–Lorentz oscillators were used in the case of the PTCA-SMs. This layered ellipsometric model is presented in Figure S4b. The effective medium approximation (EMA) model was applied for the fitting of the refractive index and extinction coefficient of the P3HT/PTCA-SMs composite [46,47] (Figure S4c). 

The general relation of EMA:

(3) $$\frac{{\tilde{n}}_{e}^{2}-{\tilde{n}}_{h}^{2}}{{\tilde{n}}_{e}^{2}+2{\tilde{n}}_{h}^{2}}=\sum_{i=1}^{N}f_{i}\frac{{\tilde{n}}_{i}^{2}-{\tilde{n}}_{h}^{2}}{{\tilde{n}}_{i}^{2}+2{\tilde{n}}_{h}^{2}}$$

where *n_e_* is the complex refractive index of the effective medium, *n_h_* is the refractive index of the host material, and *n_i_* is the refractive index of the inclusions. *N* is the number of constituents and *f_i_* is the volume fractions of inclusions. In this case, we used the Bruggeman model in which *n_h_* = *n_e_*, where P3HT and PTCA-SMs are the inclusions. In the Bruggeman model, the volume fractions of the inclusions are described by the following relation [48]:

(4) $$f_{i\;=}\frac{\varepsilon_{e}-\varepsilon_{h}}{\varepsilon_{i}-\varepsilon_{h}}\cdot \frac{\varepsilon_{i}+\varepsilon_{e}}{\varepsilon_{e}}$$

where ε_e_, ε_h_ and ε_i_ are dielectric effective permittivity, permittivity of host and included material, respectively. 

**Figure 2:**
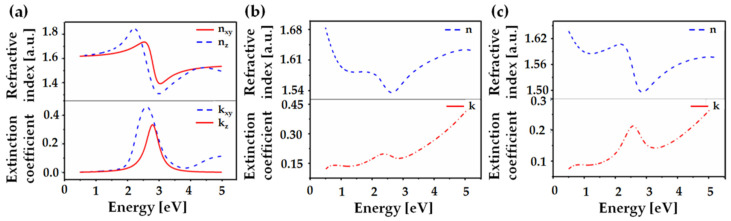
Refractive indexes and extinction coefficients of (**a**) P3HT, (**b**) PTCA-SMs, and (**c**) P3HT/PTCA-SMs.

The best fitting was achieved with a fraction of inclusion equal to *f_i_* = 0.604, i.e., for a slightly higher percentage (0.4:0.6) of PTCA-SMs than the nominal one (1:1). The optical coefficients: (refractive index n and extinction coefficient k) determined for P3HT, PTCA-SMs, and their blend are shown in Figure 2a–c. The modulus of the refractive indexes, read at 1.96 eV (632 nm), were n_xy_ = 1.67 and n_z_ = 1.76 for P3HT, n = 1.58 for PTCA-SMs, and n = 1.60 for P3HT/PTCA-SMs. 

The P3HT ellipsometry results were fitted using an anisotropic model, which showed the changes in the refractive indices between the “xy” plane and the “z” direction. This was caused by the perpendicular orientation of the polymer chains with respect to the substrate. The drop casting method strongly influenced the crystallization of the polymers and favored a greater edge-on orientation [49]. In the case of PTCA-SMs and the blend, the anisotropic model was not able to fit the ellipsometry results, which may have occurred due to the planar and agglomeration nature of PTCA-SMs in the thin film. As we can see in the AFM pictures, the PTCA-SMs agglomerate in single crystallites whose orientations are more face-on, which is more visible in the XRD results than in the ellipsometry. As an effect, we obtained the blend, whose internal morphology was a mixture of these two types of crystallization orientations, which was also better interpreted by XRD. 

**Figure 3:**
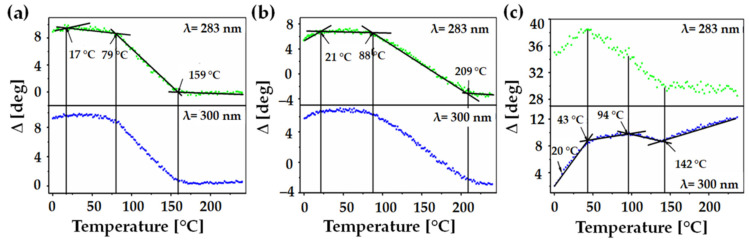
Ellipsometry temperature scans of P3HT (**a**), PTCA-SMs (**b**), and P3HT-PTCA SMs (**c**). Green and blue dots in graphs represent the ellipsometric angle ∆ at 283 and 300 nm, respectively.

**Figure 4:**
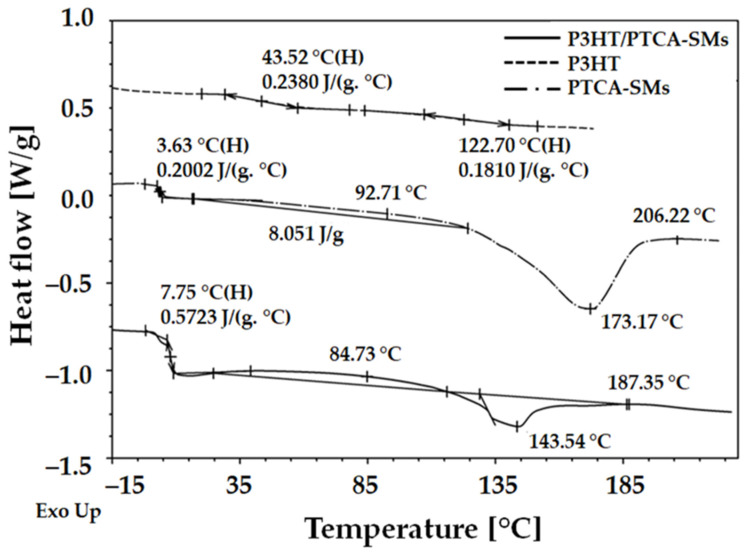
DSC plots of P3HT, PTCA-SMs, and P3HT-PTCA SMs.

To evaluate the thermal behaviors of P3HT, PTCA-SMs, and the blend of P3HT: PTCA-SMs, we performed a thermal analysis using two different techniques, variable-temperature spectroscopic ellipsometry and differential scanning calorimetry, similar to our previous works [36,37,46]. We showed the dependency of the ∆ ellipsometry angle as a temperature function for selected wavelengths. In this case, two single wavelengths, λ = 283 and 300 nm, were selected depending on the low or high dispersions of the points. The properly selected curves were fitted with straight lines using the least squares method. The values of temperature transitions were determined at the intersections of these lines. The curves for every single wavelength were obtained using a special script created by our team with spectroscopic ellipsometry software version SpectraRay3 software [50]. Every temperature scan contained data obtained from approximately 200 readings, which were taken one after the other and recorded every 10 s with increasing temperature values. The thermal analysis results obtained using the VTSE are presented in Figure 3a–c, while the results obtained with the DSC method are shown in Figure 4. It is apparent that the temperatures obtained from both techniques are quite similar; although, they do not correspond perfectly. This disparity can be attributed to the form of the material used for the examination, such as ellipsometry measured layers, while the DSC method analyzed bulk values. Several parameters, such as surface area, confinement effect, and heating rate, affected the thermal properties of these two different forms of materials [45,51]. In the case of P3HT, three characteristic temperature values were obtained from the ellipsometry scan, at around 17, 79, and 159 °C (Figure 3a). For the same material in bulk, two characteristic temperatures of about 44 and 123 °C were determined using the DSC method (Figure 4). The first temperature that was obtained under ellipsometry was the glass transition temperature (T_g_) of P3HT, which was not visible in the DSC scan. Two additional temperatures, T_CC1_ and T_CC2_, visible in the ellipsometry scan and in the DSC, corresponded to the cold crystallization of the main and side chains [52,53,54].

In the case of PTCA (Figure 3b), three temperature values were measured at 21, 88, and 209 °C via ellipsometry. We believe the first characteristic temperature, placed at around 21 °C, is the glass transition. The second temperature, placed at 88 °C, can be assigned to cold crystallization, and the temperature at around 209 °C to the melting point of the material. Using the DSC method, we obtained additional characteristic temperatures at around 3.6, 92, 172, and 206 °C. The T_g_ value recorded by the DSC method was lower than the same one recorded using ellipsometry. The second temperature, visible in the ellipsometry at 88 °C, corresponded to the first T_CC_ temperature in the DSC scan, where there was a very wide and low crystallization peak (at around 93 °C). It was noticed, in the range of 120–195 °C, two processes overlapped: still ongoing cold crystallization (T_CC_) and the melting of crystallites (T_cm_) at 172 °C. The temperature at 195 °C was the value of the end temperature of the melting peak—T_EM_—and was in very good agreement with 209 °C, recorded with ellipsometry [55]. In the case of the P3HT:PTCA-SMs blend, three characteristic temperatures were determined using ellipsometry (Figure 3c), at around 43, 94, and 142 °C. The glass transition, placed at about 20 °C, was slightly unclear. Subsequent temperatures of 43 and 94 °C could be attributed to the cold crystallization of the P3HT phase [56] and the P3HT/PTCA-SMs blend, respectively. The temperature of 142 °C was probably the melting point of the crystallites. Using the DSC scan, temperatures were recorded at around 8, 84, 143, and 187 °C. The first temperature recorded using the DSC method was T_g_, which was obtained from PTCA-SMs and P3HT, which overlapped here. The temperature at 84 °C recorded by the DSC method corresponded to the second of the two T_CC_ temperatures, recorded by ellipsometry at 94 °C. The last two temperatures of the blend, placed at around 143 and 187 °C, represented T_CM_ and T_EM_, respectively. The T_CM_ value coincided with the value recorded with the ellipsometer (Figure 3c).

**Figure 5:**
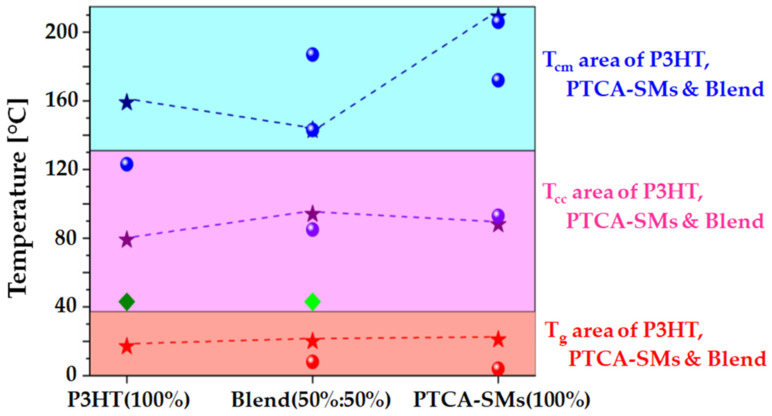
Comparison of characteristic temperatures for P3HT, PTCA-SMs, and their blend.

Based on the data obtained by the VTSE and DSC methods, we constructed a comparison graph (Figure 5) where the obtained results were presented as a function of the percentage of PTCA-SMs. The temperatures of the thermal transitions (including phase transformations (T_CC_)) for P3HT, PTCA-SMs, and their 50% blend were marked on it. We marked three areas in this case: the T_g_ area (red), the T_CC_ area (violet), and the T_CM_ area (blue). The temperatures obtained from the VTSE were marked with stars, and the temperatures obtained from the DSC method were marked with balls. Two additional temperatures, very clearly observed by the DSC and ellipsometry methods, were marked with green squares. The temperature of 43 °C, corresponding to T_CC_ P3HT [41], detected by DSC, was also detected using ellipsometry, in the case of a temperature scan of the mixture. The presence of two T_CC_ temperatures at 43 and 94 °C meant that, in the P3HT:PTCA-SMs layer, apart from the crystalline phase of the mixture, there was also a crystalline phase coming from P3HT.

The greatest difference between the results obtained from the VTSE and DSC methods can be observed in the case of the polymer. When P3HT was deposited as a layer on a substrate, the polymer molecules arranged themselves into a cohesive and moderately interconnected network, forming a matrix-like structure. In the case of PTCA-SMs, the layer was formed as a result of the particles’ agglomeration onto the substrate. The agglomeration already started in solution, and the evaporation of the solvent made this process easier.

**Figure 6:**
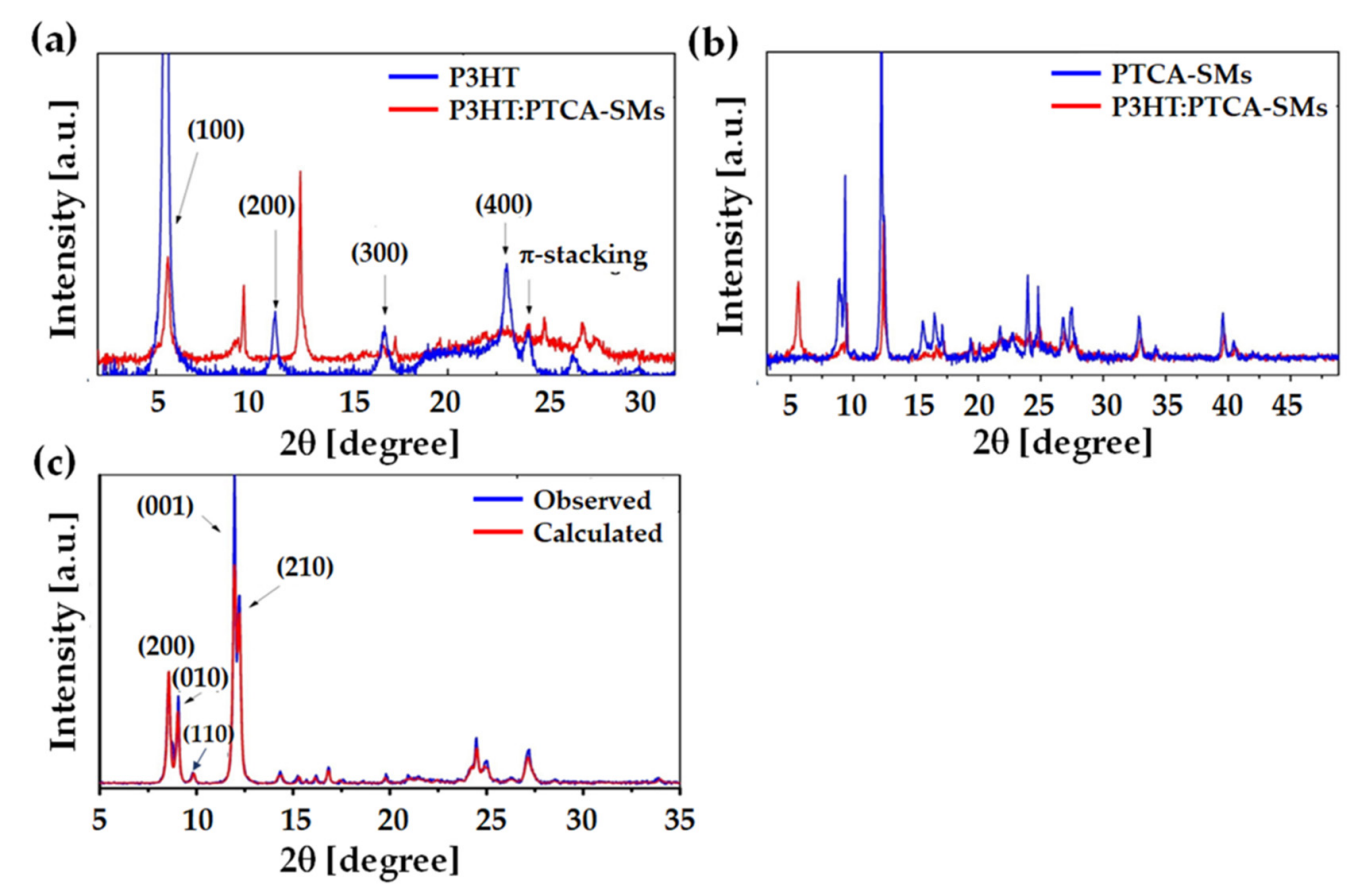
XRD analysis: (**a**) XRD patterns of P3HT and P3HT:PTCA-SMs-blend films; (**b**) XRD patterns of PTCA-SMs and P3HT:PTCA-SMs-blend films; and (**c**) comparison of the calculated and observed XRD patterns of PTCA-SMs.

The distribution of molecules in the unit cell of crystallites and the orientations of the P3HT and PTCA-SM crystallites in the examined films were determined by XRD (Figure 6). As mentioned in the experimental section, the XRD measurements were performed using the Bragg–Brentano geometry. Such an experimental configuration resulted in the observation of reflections only from crystallites with crystallographic planes arranged parallel to the surface of the sample. Thus, taking into account Bragg’s law, λ = 2 dsin(θ), where λ is the X-ray wavelength, d is the interplanar distance, and θ is the angle of incidence of the X-rays, we can determine the characteristic distance corresponding to the peak at position 2θ on the XRD scan, in crystallites with atomic planes parallel to the surface of the sample. This knowledge empowers us to derive significantly more information from the XRD scans when we possess information regarding the spatial distribution of atoms or molecules within the unit cell. Specifically, let us consider the scenario where we can pinpoint a peak on the XRD scan that aligns with the distinctive spacing between planes of the π-stacked aromatic rings. Hence, armed with the knowledge about the molecular distribution within the unit cell, as demonstrated in our PTCA-SMs case, we can confidently assure that it originates from crystalline structures, which also yield reflections from the (001) planes. This allows us to conclude that the π-stacking configuration within these crystallites assumes a face-on arrangement. Furthermore, the presence of reflection peaks on planes with (h00) or (0k0) indices, where “h” and “k” are natural numbers, provides insights into the arrangement of π-stacked aromatic rings within the originating crystalline structures. For instance, in the discussed context, the presence of the reflection identified as (010) implies the edge-on configuration of PTCA-SMs within the crystallites contributing to this peak. Having addressed these pivotal considerations related to the foundational underpinnings of our conclusions, we can proceed to present and discuss the acquired XRD results. 

X-ray examinations were conducted on layers consisting of P3HT, PTCA-SMs, and a 1:1 blend of both materials. To enhance the comprehension of XRD patterns and variations in the molecular alignment within the P3HT:PTCA-SMs layers, we presented and analyzed the XRD scans in pairs. Specifically, Figure 6a displays the XRD pattern for the P3HT layers and the P3HT:PTCA-SMs mixture, while Figure 6b showcases the XRD results for the PTCA-SMs nanoparticles and the P3HT:PTCA-SMs-mixture layers. In the polymer-layer scan, the presence of crystallites exhibiting a mixed orientation is evident. This interpretation is facilitated by the fact that the XRD scan of the P3HT layer, as presented in Figure 6a, distinctly exhibits Bragg reflections originating from the family of lattice planes (h00), where h = 1, …, 4. The most prominent peak, denoted as (100) at 2θ = 5.6°, signifies a lamellar arrangement of the alkyl segments within the P3HT chain, with a characteristic spacing of 15.1 Å. Additionally, the other three peaks observed at approximately 11, 16, and 23 degrees in 2θ angles correspond to the previously mentioned peaks for “h” values of 2, 3, and 4. Furthermore, the presence of the (010) peak at 2θ = 24.15° signifies an interplanar distance of 3.68 Å, which is characteristic of the π-stacking of the thiophene rings within the main chain of P3HT. Simultaneously, this reflection reveals the face-on arrangement of the thiophene rings within the crystalline structures of the P3HT layer. 

Taking into account that lamellar stacking and π–stacking are orthogonal, we can infer that the P3HT crystallites within the tested P3HT layer exhibit a mixed orientation. It is worth emphasizing that the identification of peaks in the XRD pattern for the P3HT layer closely and quantitatively aligned with the values reported in the literature [57,58,59]. Subsequently, when we compared the XRD scans of the polymer and P3HT:PTCA-SM-mixture layers (Figure 6a), it became evident that nearly all of the polymer peaks were present in the blend-layer scan. This observation suggests the similar, namely mixed, arrangement of P3HT crystallites within this layer. It is worth noting that the XRD scan for the PTCA-SM nanoparticle layer revealed a relatively abundant number of peaks, signifying a high level of crystallinity within this layer [60]. 

To achieve greater insights into the microstructure of the PTCA-SMs layer, we conducted semi-empirical calculations using MOPAC2016 [61] to model the distribution of PTCA-SMs within the unit cells of the crystallites. The calculations proceeded as follows: in the initial step, we selected unit cells that were best suited for XRD scanning based on the analysis of the XRD data using version Expo2014 software [62]. In the subsequent step, we employed the MOPAC program, specifically at the PM6-D3H4 [63] calculation level, which incorporated PM6 with corrections for dispersion interactions (D3) and hydrogen bonds (H4), to optimize the distribution of PTCA-SMs molecules within pre-defined unit cells. Following this, we conducted a full optimization of the examined systems, eliminating constraints on the parameters of elementary cells. Among the systems explored, the one with the lowest energy value, characterized by a heat of formation of −590.2 kcal/mol and the unit cell parameters, a = 20.92 Å, b = 9.85 Å, c = 7.53 Å, α = 95.6 degrees, β = 81.2 degrees, and γ = 88.9 degrees, was selected. Subsequently, matching to the observed XRD pattern was performed using Expo2014. Notably, Rietveld’s and Le Bail’s refinement for unstructured parameters was applied in this process. The unit cell parameters obtained in this final step remained largely unchanged.

The Miller indices of the primary peaks are denoted in Figure 6c. It is important to highlight that the unit cell parameters for PTCA-SMs crystallites, as determined in our study, closely aligned with the values reported in [58,59,60], taking into account that our determined cell contained one additional molecule. Figure 6c compares the computed XRD scan with the observed one, while Figure S5 illustrates the distributions of PTCA-SMs within the determined unit cells. Notably, it becomes evident that within the unit cell derived from the XRD pattern-matching stage, PTCA-SMs exhibited a larger degree of overlap. Furthermore, we calculated the crystallite size using the double-Voigt method in the Topas program, as outlined in the Topas Technical Reference Manual by Bruker. Specifically, the volume-weighted crystallite dimension (Lvol), which was oriented perpendicular to the sample’s surface, was obtained by performing Lorentz and Gaussian convolutions on the integral breadth. Our XRD scans of P3HT revealed an Lvol of 35.6 nm for the (100) peak. However, when P3HT was mixed with PTCA-SMs, the corresponding crystallite size increased to 55.9 nm. Notably, a similar trend in crystallite sizes was observed in PTCA-SMs layers and their mixtures with P3HT. For instance, the Lvol associated with the (001) peak in SM XRD was 30.2 nm, whereas in the mixture, it increased to 43.1 nm [64]. 

**Figure 7:**
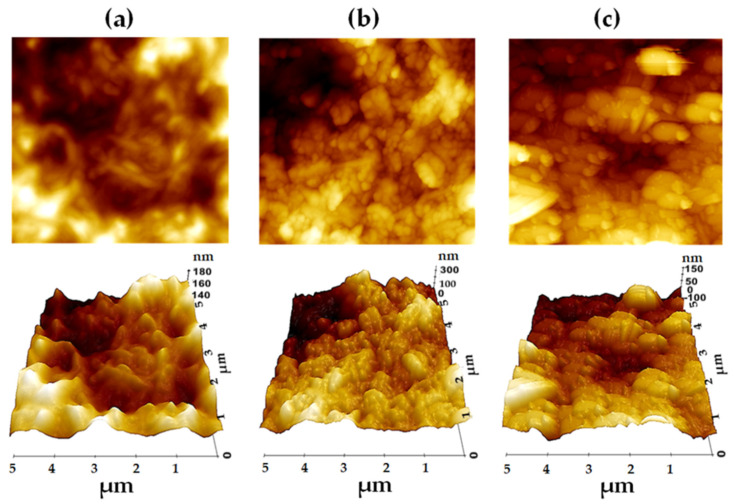
AFM 5 × 5 µm 2D and 3D topographic surface images of (**a**) P3HT, (**b**) PTCA-SMs, and (**c**) P3HT:PTCA-SMs.

The image with surface morphologies of P3HT, PTCA-SMs, and P3HT:PTCA-SMs films were taken on annealed films and deposited onto microscopic glass using optical microscopy and AFM (Figure 7 and Figure S7). Figure 7a–c shows 5 × 5 µm; the surface morphologies of P3HT, PTCA-SMs, and the blend were obtained by AFM in the noncontact mode. The topography of P3HT (Figure 7a) seems to be rather disordered with a nanofibrillar structure. The surface is not smooth and there are visible irregularities, where the difference between the lowest and highest points is in the range of 40–160 nm with a surface r.m.s roughness of ~11.61 nm. A large agglomeration is visible in the PTCA-SMs surface topography resulting in a higher surface root mean square (r.m.s) roughness of ~99.12 nm. The formation process of the layer had a notable impact on the surface topography. Drop-casting was employed to prepare the sample, which led to PTCA-SMs readily agglomerating, as is evident in the images (Figure 7b). In contrast, the layer of the P3HT:PTCA-SMs blend exhibits a higher degree of order and regularity, with a lower surface r.m.s roughness of approximately 26.45 nm (Figure 7c). This surface enters an intermediate state when compared to the topography of the pure materials. There are quite long crystallites visible, with sizes in the range of 0.5–1.2 µm (Figure S7).

Long crystallites were formed as a result of the intermolecular interaction between both pure materials as well as the annealing temperature (200 °C) and the use of a binary solvent (CHCl_3_ and DMF). The temperature and the binary solvent also played a significant role in effectively controlling the organic semiconductor crystallization, thin-film morphology, as well as and crystal orientations, as depicted in Figure S7 [6]. Higher temperatures promoted the dissolution of solutes in the solvent, increasing their mobility and aiding in crystal growth [65,66,67]. A binary solvent system, such as the combination of CHCl_3_ and DMF, modified the evaporation rate of individual solvents, thereby exerting control over the nucleation and growth of the crystals. This dual-solvent approach is particularly valuable when one solvent is well-suited for a specific component while not as effective for another. The unique interactions between the solute and solvents promoted the formation of highly organized crystalline structures within the thin films [68,69].

**Figure 8:**
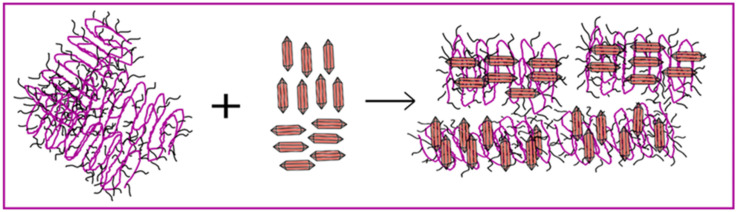
Ordering of P3HT/PTCA-SMs crystallites inside the thin film.

Figure 8 illustrates the arrangement of the crystallites in the thin film. Based on the XRD results, the synthesized P3HT spectrum exhibits twice as many peaks as the commercially available P3HT. This suggests a higher degree of lamella packing and an enhanced interlocking of aliphatic chains, indicative of a well-ordered polymer structure. In the spectrum of pure PTCA-SMs, it can be observed that the planes of small molecules align either perpendicularly or parallel to the substrate. The PTCA-SMs likely integrate between the P3HT crystal lamellae, facilitating the formation of relatively long crystallites, as indicated by the AFM analysis. This resulted in the arrangement of the P3HT:PTCA-SMs combination of edge-on and face-on structures, with this arrangement being forced mainly by the incorporation of large particles of PTCA-SMs. 

## 4. Conclusions

In this article, our research delved into the examination of the physical properties of blend films comprising P3HT/PTCA-SMs, drawing comparisons with the pristine P3HT and PTCA-SMs films. Our primary emphasis was on conducting a comprehensive analysis encompassing the optical, thermal, crystalline, and surface morphological characteristics of these samples in thin films. Our findings demonstrated that the resulting film blend was a homogenous material, featuring an additional crystallized phase of P3HT. This was confirmed by optical studies, where a considerable difference was observed between the shape and intensity of the refractive indices of P3HT, PTCA-SMs crystallites, and their blend layers. Moreover, DSC and VTSE studies on these films indicated a single glass transition temperature (T_g_), suggesting the good dispersion of PTCA-SMs within the P3HT polymer matrix. Furthermore, in the P3HT:PTCA-SMs blend, we observed the presence of two cold crystallization (T_CC_) temperatures. Intriguingly, the first temperature aligned precisely with the T_CC_ of P3HT, suggesting the simultaneous coexistence of two crystalline phases: P3HT and the P3HT:PTCA-SMs blend. The existence of these crystallized phases was also confirmed through AFM and X-ray measurements. In addition, based on ellipsometric modeling, it was determined that the percentage content of PTCA in the tested blends was slightly higher than that of P3HT, which certainly influenced the ease of crystal formation on the film surface. AFM microscopy corroborated the presence of relatively substantial crystallites, with lengths falling within the range of 0.5–1.2 µm, visible on the surface of the samples. Based on the obtained XRD results, it is shown that the microstructure of the obtained blend films is a combination of the edge-on arrangement of P3HT crystallites with PTCA-SM crystallites with preferentially oriented (001) planes parallel to the surface layer, which indicates the potential application of this material as an active layer in optoelectronic devices. Thus, our future study will focus on modulating the energy gap of the blend by varying the percentage of P3HT relative to PTCA-SMs, evaluating their electrical properties, and constructing simple devices.

## Data Availability

Data presented in this article are available on the CMPW-PAN website.

## Supplementary Materials

The following supporting information can be downloaded at: https://www.mdpi.com/article/10.3390/nano13222918/s1, Figure S1. 1H nuclear magnetic resonance (NMR) of P3HT; Figure S2. Fourier-transform infrared (FT-IR)—ATR of PTCA-SMs; Figure S3. Energy gap of PTCA-SMs with different thickness of thin films; Figure S4. Ellipsometric Models of (a) P3HT, (b) PTCA-SMs, and (c) P3HT: PTCA-SMs; Figure S5. Comparison of PTCA-SMs distribution in elementary cells determined based on semi-empirical calculations using (a) MOPAC2016 and matching to the X-ray pattern with (b) EXPO2014 software; Figure S6. The mean square root of the roughness of tested samples; Figure S7. 1.25 × 1.25 and 0.7 × 0.7 topographic 3d images of crystallites visible in P3HT: PTCA-SMs blend surface. Ref. [70] is cited in Supplementary Materials.
