# Supplementary material for: Exploring the Influence of P3HT on PTCA Crystallization and Phase Behavior in Thin Films
Source: Nanomaterials (Basel). 2023 Nov 8;13(22):2918. doi: 10.3390/nano13222918 (PMC10675274; doi:10.3390/nano13222918)
Supplement: Supplementary file 1 [file nanomaterials-13-02918-s001.zip › nanomaterials-2665575-supplementary.pdf]

Supplementary Materials

Exploring the Influence of P3HT on PTCA Crystallization and Phase Behavior in Thin Films

Pallavi Kumari[1*], Barbara Hajduk[1], Henryk Bednarski[1,], Paweł Jarka[2], Henryk Janeczek[1] , Mieczysław Łapkowski[1,3]

1 Centre of Polymer and Carbon Materials, Polish Academy of Sciences, 34 Marie Curie-Skłodowska str., 41-819 Zabrze, Poland.

2 Department of Engineering Materials and Biomaterials, Silesian University of Technology, 18a Konarskiego str., 41-100 Gliwice, Poland.

3. Department of Physical Chemistry and Technology of Polymers, Silesian University of Technology, Faculty of Chemistry, M. Strzody 9, 44-100 Gliwice, Poland

* Correspondence: pkumari@cmpw-pan.pl

Citation: To be added by editorial staff during production.

Academic Editor:

Received: date

Revised: date

Accepted: date

Published: date

Copyright: © 2023 by the authors. Submitted for possible open access publication under the terms and conditions of the Creative Commons Attribution (CC BY) license (https://creativecommons.org/licenses/by/4.0/).

1. Results

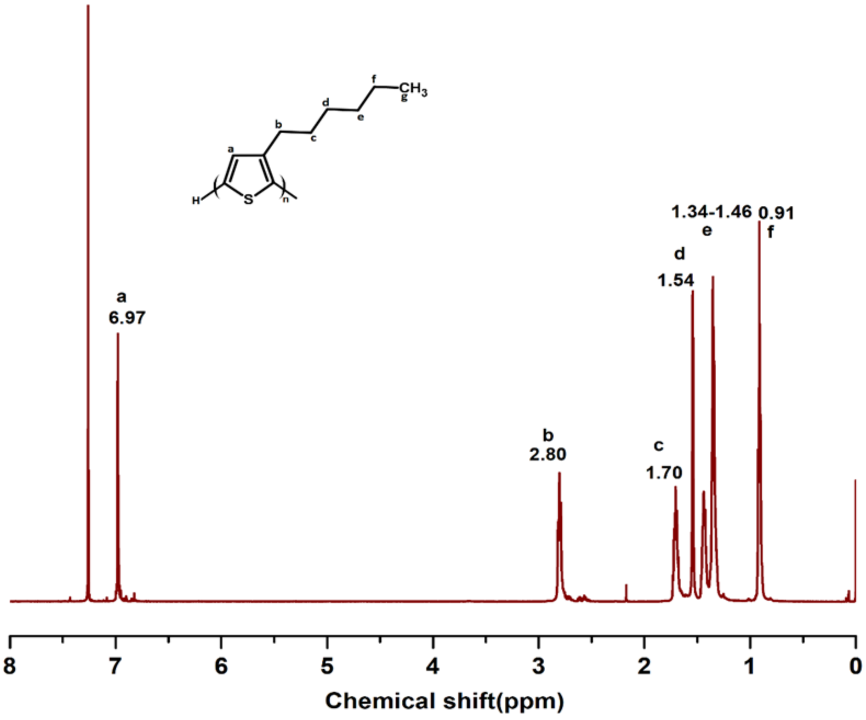

Figure S1. 1H nuclear magnetic resonance (NMR) of P3HT.

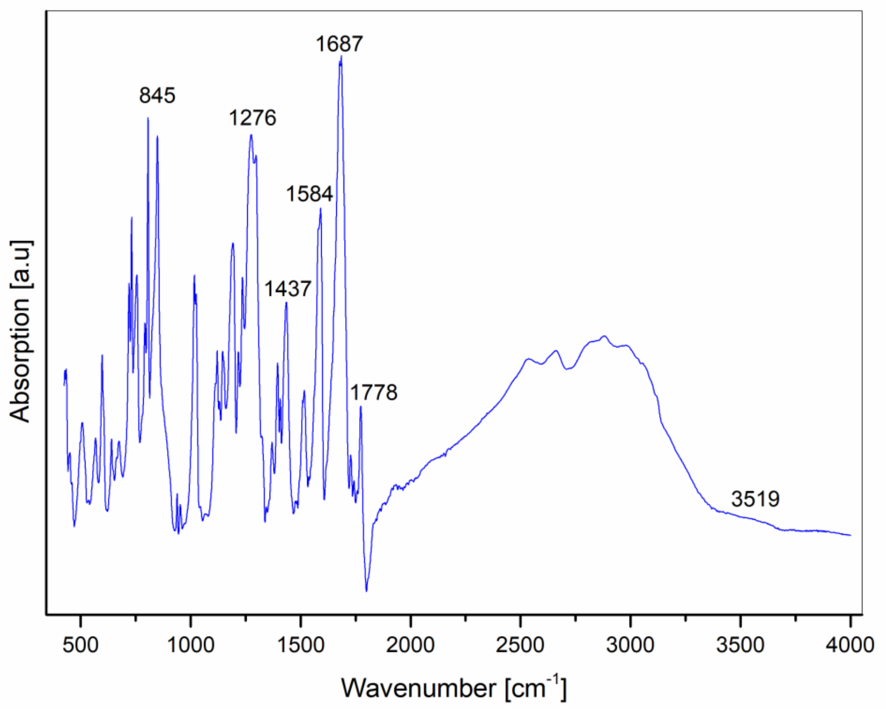

Figure S2. Fourier-transform infrared (FT-IR)–ATR of PTCA-SMs.

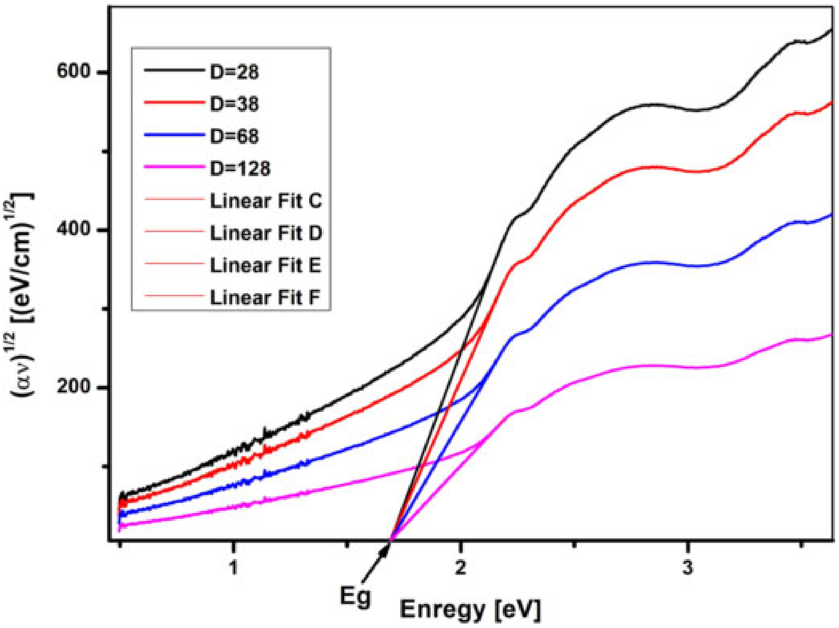

Figure S3. Energy gap of PTCA-SMs with different thickness of thin films.

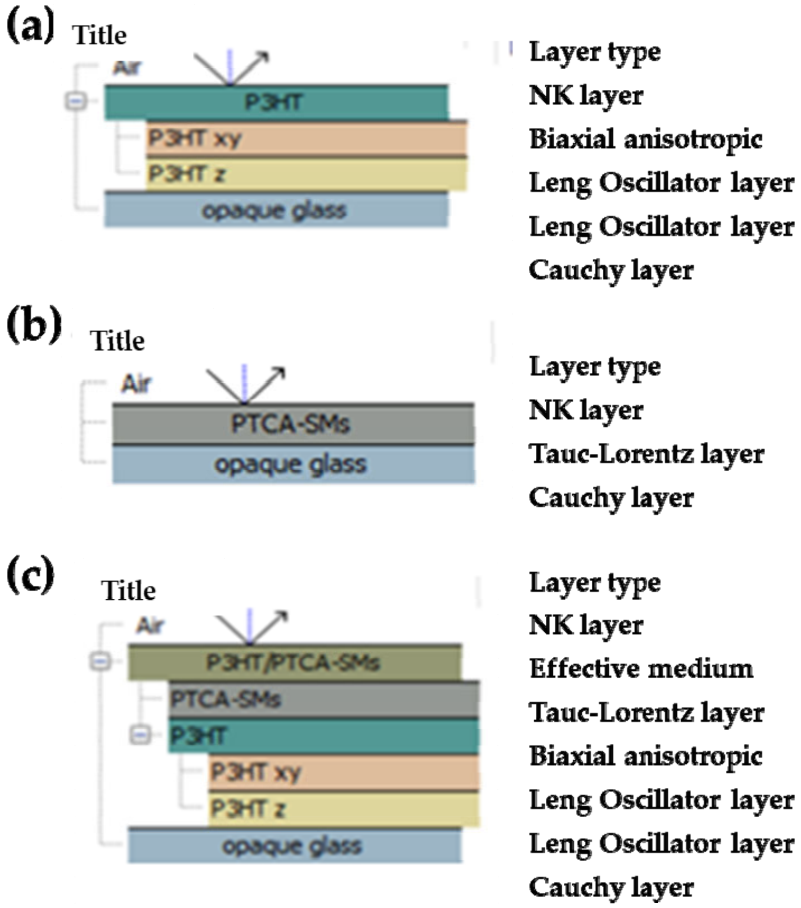

Figure S4. Ellipsometric Models of (a) P3HT, (b) PTCA-SMs, and (c) P3HT: PTCA-SMs.

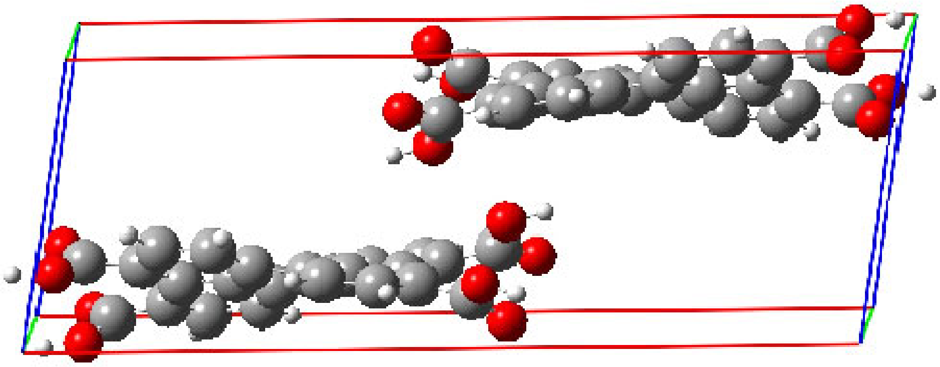

a)

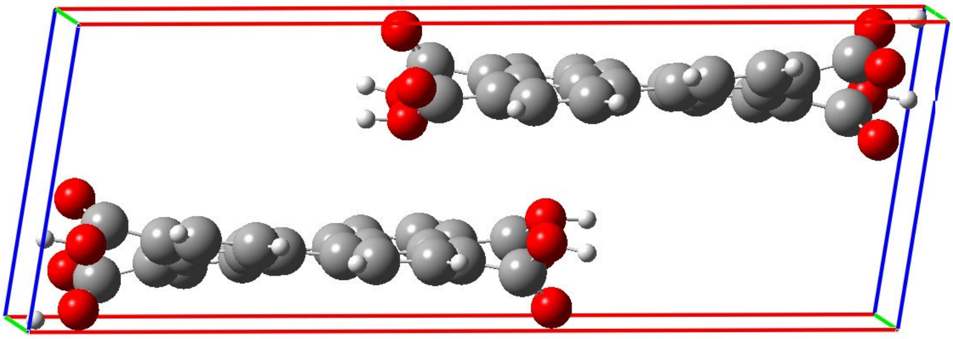

b)

Figure S5. Comparison of PTCA-SMs distribution in elementary cells determined based on semi-empirical calculations using (a) MOPAC2016 and matching to the X-ray pattern with (b) EXPO2014 software.

Surface roughness calculation

The root mean square roughness - R_q was applied for surface characterization of investigated samples. The R_q is defined with the following equation:

$$R_q = \sqrt{\frac{1}{m}\sum_{i=1}^{m}(Z_i - \overline{Z})^2},$$

where the m parameter is number of sampled points, Z_i is height of individual point and $\overline{Z}$ is average height value [1]. R_q was determined for three surface sizes: 2 × 2, 5 × 5 and 20 × 20 µm and these values are showed in graph E1 below:

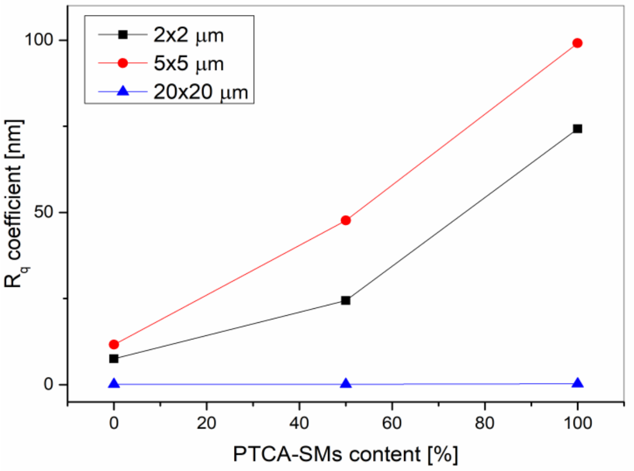

Figure S6. The mean square root of the roughness of tested samples.

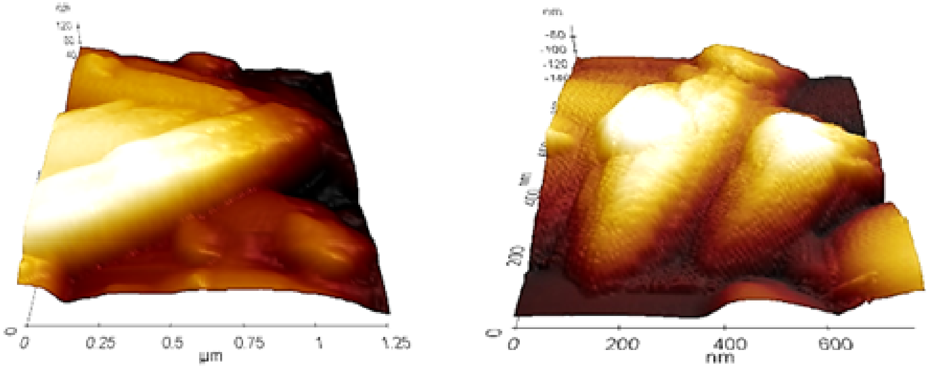

Figure S7. 1.25 × 1.25 and 0.7 × 0.7 topographic 3d images of crystallites visible in P3HT: PTCA-SMs blend surface.

References

[1] Borgesi, A.; Tallarida, G.; Amore, G.; Cazzaniga, F.; Queirolo, F.; Alessandri, M.; Sassela, A. Influence of roughness and grain dimension on the optical functions of polycrystalline silicon films. *Thin Solid Film* **1998**, *313-314*, 243–247. DOI: 10.1016/S0040-6090(97)00826-2.
